# Building a FAIR image data ecosystem for microscopy communities

**DOI:** 10.1007/s00418-023-02203-7

**Published:** 2023-06-21

**Authors:** Isabel Kemmer, Antje Keppler, Beatriz Serrano-Solano, Arina Rybina, Buğra Özdemir, Johanna Bischof, Ayoub El Ghadraoui, John E. Eriksson, Aastha Mathur

**Affiliations:** 1 Euro-BioImaging ERIC Bio-Hub, European Molecular Biology Laboratory (EMBL) Heidelberg, Meyerhofstraße 1, 69117 Heidelberg, Germany; 2 Euro-BioImaging ERIC Statutory Seat, Tykistökatu 6, P.O. Box 123, 20521 Turku, Finland

**Keywords:** Bioimaging, FAIR, Community, Metadata, Data management

## Abstract

Bioimaging has now entered the era of big data with faster-than-ever development of complex microscopy technologies leading to increasingly complex datasets. This enormous increase in data size and informational complexity within those datasets has brought with it several difficulties in terms of common and harmonized data handling, analysis, and management practices, which are currently hampering the full potential of image data being realized. Here, we outline a wide range of efforts and solutions currently being developed by the microscopy community to address these challenges on the path towards FAIR bioimaging data. We also highlight how different actors in the microscopy ecosystem are working together, creating synergies that develop new approaches, and how research infrastructures, such as Euro-BioImaging, are fostering these interactions to shape the field.

## Introduction: The hidden potential and current challenges of bioimaging data

In recent years, the field of bioimaging has evolved from its qualitative origins, mainly focusing on the visualization of individual biological processes and structures, to a highly quantitative discipline, producing large and complex, sometimes multimodal, datasets requiring sophisticated and robust computational and statistical methods for analysis. At the same time, imaging is becoming a key enabling technology in the biological and biomedical sciences and beyond, as evidenced by an incredibly wide range of imaging modalities and techniques that are constantly being developed (Ouyang and Zimmer 2017). Bioimaging methods now span length scales from single molecules to cells, all the way to entire multicellular organisms, as well as time scales ranging from fractions of milliseconds to days. Additionally, multiplexing allows the simultaneous visualization of a multitude of sample characteristics in parallel (Ellenberg et al. 2018). The underlying technical and, more importantly, computational advancements are now enabling scientists to understand and map spatiotemporal biological processes in unprecedented quantity and detail.

While this diversity demonstrates the immense potential of bioimaging, it also results in larger and more complex datasets that present many new challenges. Thus, we are now witnessing the transformation of bioimaging into the era of big data, where data requires rigorous standards for metadata, data management and dissemination procedures than ever before (Driscoll and Zaritsky 2021). Given the rising cost and time involved in increasingly intricate, including correlative and multimodal imaging experiments and analysis, it is imperative to maximize the reliable use and potential reuse to increase the value output of microscopy data. While imaging has the inherent potential to become increasingly impactful to study processes on molecular, cellular, and organism level in vivo and in vitro*,* its full potential can only be realized and effectively exploited when these challenges are adequately addressed.

This goal can be achieved by adhering to the FAIR principles, which provide guidelines to improve the **F**indability, **A**ccessibility, **I**nteroperability, and **R**eusability of data so that it can be shared in a way that enhances and promotes reuse by both humans and machines (Wilkinson et al. 2016). In short, according to the FAIR principles, (i) each data element should have a unique identifier associated with searchable metadata to be findable; (ii) data should be accessible and retrievable through well-defined and standardized access protocols; (iii) data and metadata should be described using widely adopted and domain-relevant vocabularies and ontologies to be interoperable; and (iv) data should be described with rich metadata and cross-references to other data sources and publications as well as state a clear license for reuse.

Since their formal statement in 2016, the FAIR principles have become a cornerstone for making research output more impactful and usable, with growing adoption by scientific communities. Research communities are at different stages along the spectrum of FAIR data: Some domains have been implementing similar principles long before the advent of ‘FAIR’. Examples of communities with well-established FAIR standards include structural biology, genomics, and astronomy, among others (Pepe et al. 2014; Morris 2018; Berman et al. 2020; Byrd et al. 2020). While some research communities are just starting their FAIR journeys, others, such as bioimaging, have been experiencing a shift in mindsets towards FAIR over the past years. On this path, the imaging community has made significant efforts, but also faces considerable difficulties due to a large diversity of methods, research questions, and produced data, which are currently being addressed with various solutions that will be outlined in this commentary. Although the FAIR principles equally apply to both sensitive and Open data, within the scope of this work we will elucidate current state and undertakings in the field of Open microscopy image data, acknowledging the potential for interoperability between microscopy and medical imaging fields. Overall, we believe that realizing the FAIR vision will require both concrete support and incentives from an ecosystem of diverse stakeholders: researchers, imaging facilities, universities, policy makers, funders, journals, communities, and instrument manufacturers, among others, working together towards the same goal. By linking communities across scientific areas and disciplines and fostering exchange of knowledge and know-how on an international level, research infrastructures like Euro-BioImaging (see Box 1) are a central player within that landscape. In this way, bioimaging will move to a place where data and software sharing according to the FAIR principles is a top priority, laying the foundation for accelerated innovation and cutting-edge research.

Box 1: Realizing the FAIR vision through European Research Infrastructures Research Infrastructures^1^ refer to facilities, services, and resources of high-quality standards and of national and international significance that are open to the scientific community. Due to their long-lived nature, they are particularly conducive to knowledge and technology transfer and foster links and collaborations with all their stakeholders, including academia, industry, and national and international authorities. As one of these, Euro-BioImaging ERIC^2^ is a European Research Infrastructure that democratizes access to world-class imaging services ranging from nano to macroscopic scales (Pfander et al. 2022). As of March 2023, it provides open access to 105 different state-of-the-art biological and biomedical imaging technologies in 173 imaging facilities organized in 35 Nodes hosted by national research institutions and universities across Europe. Sixteen European countries and the international organization EMBL (European Molecular Biology Laboratory) have committed to jointly operate this pan-European research infrastructure. This tremendous support at the European level makes the infrastructure a unique resource for researchers, even on the global landscape. All scientists, regardless of country, affiliation, research area, or expertise, can benefit from these high-quality services provided by leading imaging facilities including a network of experts. In addition, researchers have access to highly specialized training, much-needed data management and image analysis services to help them to extract meaningful information from bioimaging data. In this way, Euro-BioImaging promotes collaboration, innovation, sharing of resources and expertise as well as Open Science and Open Access in the field of life science imaging, with the goal of advancing scientific knowledge and driving technological innovation.

## Stepping stones on the road to FAIR

**Fig. 1:**
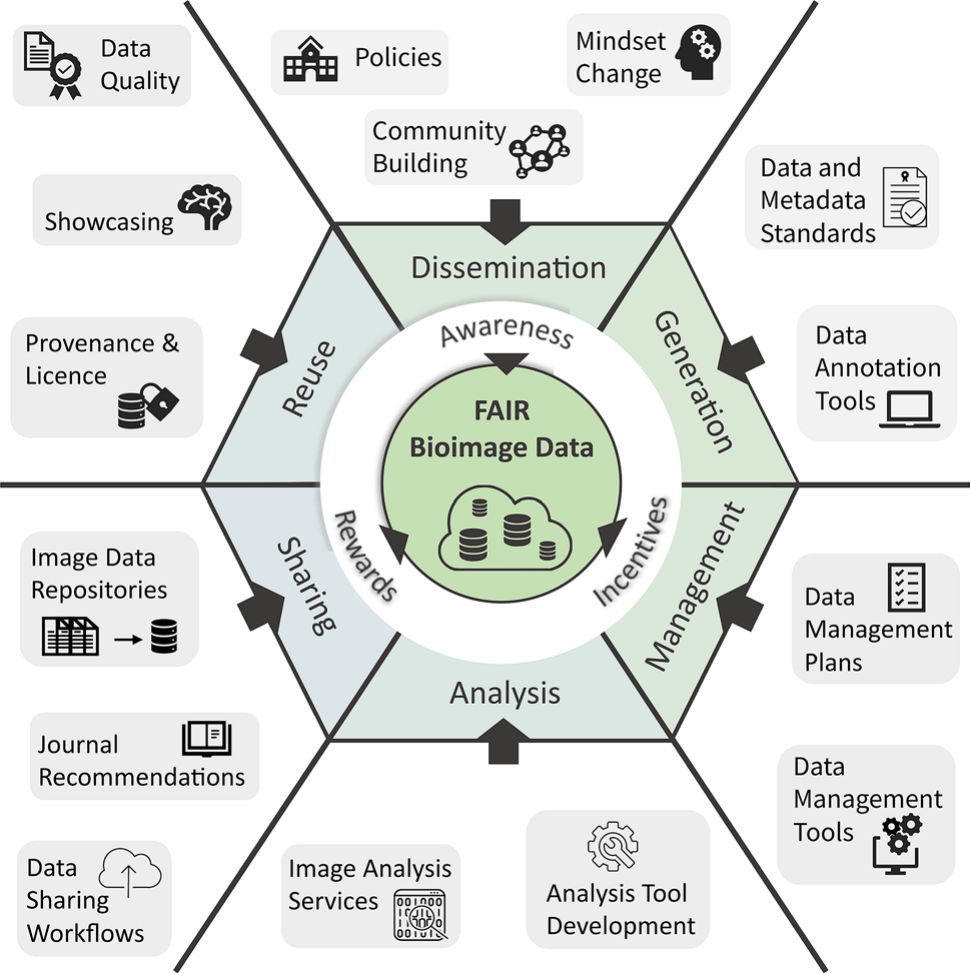
Six facets of FAIR bioimaging data (data generation, data management, data analysis, data sharing, data reuse, and FAIR dissemination) alongside the approaches and tools currently under active development by the bioimaging community. Further improving all of these areas still requires three overarching elements (awareness, incentives, and rewards) to move toward the ultimate goal of FAIR bioimaging data

In contrast to more homogeneous disciplines that have a tradition of implementing FAIR principles and data sharing, bioimaging is a much more heterogeneous field with variable methodologies and protocols for preparing samples, acquiring, and analyzing data. The path of other heterogeneous disciplines to FAIR data, such as systems biology (Wittig et al. 2017), can only serve as a starting point and a source of tools and strategies rather than precise instructions. This stems from the fact that each heterogenous discipline faces individual, domain-specific problems, such as large data sizes for bioimaging, that require tailored solutions. However, based on these experiences from other domains, we can identify several obstacles that currently impede FAIR bioimaging data (Fig. 1), and devise a multifaceted approach to promote data sharing and reuse at scale (Bagheri et al. 2022). Although it may not be possible to fully adopt currently available strategies and pipelines, the bioimaging community can still draw inspiration from them to shape the path of microscopy towards FAIR data.

### Facet 1: Image data generation and metadata

Annotating data with descriptive, high-quality metadata is a prerequisite for almost all aspects of FAIR data. Thus, imaging datasets become fully valuable and usable only when accompanied by rich metadata (Huisman et al. 2019). Metadata is a set of data that provides information about the original data and should include all information needed to enable discovery, interpretation and re-use of that data including, for example, data type, origin, organization, relationships to other data and more. However, there are currently still significant gaps in how complete and accurate microscopy experiments are documented and reported upon publication (Marqués et al. 2020). One reason for this reporting gap is the lack of clear recommendations and implemented standards on what metadata should be reported alongside an imaging experiment. Frequently, journals request that the description of an experiment must allow for replication of the results, which can lead to a rather vague interpretation by a large proportion of authors and data owners.

Providing concrete metadata recommendations is not an easy task. Especially when considering the wide variety of microscopy methods, it is not feasible to define concrete requirements for all existing imaging modalities and their sub-variants. In addition, the amount and type of information needed for reuse depends on the further scientific application and the needs of other broad or targeted user communities. Therefore, the bioimaging community has come together to converge on an initially flexible, high-level framework of metadata reporting guidelines that can be progressively tightened and specified by consensus of different sub-domains. These "REcommended Metadata for Biological Imaging'' (REMBI) guidelines (Sarkans et al. 2021) provide metadata aspects in eight categories ranging from the imaging data itself to metadata describing the study and the sample used that should accompany any imaging dataset. Additional detailed metadata models developed by the community exist, such as OME (Goldberg et al. 2005) and 4DN-BINA-OME (Hammer et al. 2021), but they require dedicated and sometimes extensive effort to meet the requirements and therefore lack widespread adoption. While adherence to these standards may not automatically lead to a fine-grained understanding of the imaging experiment or ensure full reproducibility, it is certainly a step in the right direction.

Euro-BioImaging advocates for the adoption of clear metadata standards and recommends consistent metadata reporting in general as well as by supporting repositories and journals. Some journals like the journal of Histochemistry and Cell Biology,^3^ or EMBO journals,^4^ provide their own metadata reporting checklist for imaging studies to try to reduce the metadata reporting gap. Others are working with community initiatives for quality assessment and quality control such as QUAREP-LiMi (Boehm et al. 2021) to propose a set of minimum microscopy metadata keywords that should always be reported at publication. Ultimately, journals are an important lever to incentivize the field to move towards more consistent metadata reporting, thereby increasing the credibility and usability of microscopy data. However, one must take care to not create too many different requirements, as this will result in considerable heterogeneity in reporting standards and thus reluctance on the part of the researcher to collect metadata. Standards should therefore be consolidated, unified, and agreed upon by the community. In this way, scientists can consider them and already comply with them before generating data, thus improving the way research is conducted long before publication.

It is also important to bear in mind that the burden and workload of recording metadata should not fall solely on the shoulders of individual researchers. The desire for comprehensive and rich metadata must therefore be carefully weighed against the additional effort and time it requires in practice. Hence investing in tools for automatic and standardized harvesting of metadata from imaging instruments, preferably at the time of image acquisition itself, is beneficial for the community. Several open-source tools for coupling image acquisition with metadata capture are currently under active development in the imaging community. These include the Micro-Meta App (Rigano et al. 2021), a tool to collect microscope metadata according to the 4DN-BINA-OME metadata standard (Hammer et al. 2021); MDEmic (Kunis et al. 2021), a metadata annotation tool; MethodsJ2 (Ryan et al. 2021), a tool to create comprehensive methods sections for publications.

Alongside developing these accessible tools and pipelines it is essential to establish over time a community-wide network of experts to advise and practically consult researchers on the implementation of these solutions. Euro-BioImaging supports its users and imaging facilities by offering guidance on how to properly—and eventually automatically—record and report metadata using appropriate tools such as the ones described above. On the other hand, microscope manufacturers also play a crucial role in the dissemination of image acquisition tools and software and work towards the development of better and more intuitive and automated metadata acquisition. Research infrastructures are in close contact and exchange with the industry partners, for example through the Euro-BioImaging Industry Board,^5^ and aim to align the efforts of the academic communities with those of the manufacturers.

Experience in other domains, such as the omics (Chervitz et al. 2011) and plant phenotyping (Papoutsoglou et al. 2020) fields, has taught us that some degree of standardization is essential for data to be truly interoperable and reusable. Therefore, it is not only important to capture metadata, but also to ensure that different metadata schemas are interoperable, i.e., they need to speak the same metadata language or at least be translatable. In this way, metadata ideally identifies relevant (biological) entities by means of controlled vocabularies and ontologies, for example using FBBI or EDAM-BioImaging (Kalaš et al. 2019). We recommend their use and provide targeted guidance for research conducted using Euro-BioImaging access as well as in the collaborating bioimaging repositories.

The COVID-19 pandemic revealed a critical lack of metadata interoperability, especially between distant research domains. To address this, Euro-BioImaging is actively shaping Horizon Europe (HE)-funded projects such as BY-COVID^6^ and EOSC4Cancer,^7^ which aim to make COVID and cancer-related data accessible to everyone and to harmonize metadata standards so that data are interoperable across disciplines. With the goal of accelerating and improving the preparedness for future pandemics and combating acute diseases of concern, these cross-disciplinary initiatives bring together key Research Infrastructures providing relevant services. Together they aim to develop and integrate common operational principles and strategies, from data collection to harmonization of and access to different types of data and metadata as done, for example, in the BY-COVID project (Hermjakob et al. 2022). The interdisciplinary nature of these HE-funded projects exposes its partners to a diverse range of communities, like biomolecular science, health science, and social science, where Research Infrastructures form valuable connections to exchange experiences on harmonizing different metadata standards. The diversity in type and accessibility of data pushes for use of common discoverability mechanisms, for instance by using catalogs like DCAT^8^ or BioSchemas.^9^ Mapping of bioimaging metadata models to these catalogs is being developed by the community. The urgent need for data stewardship to advise and assist on metadata types and standards, as well as the resources to provide data stewardship, is becoming increasingly evident and recognized as one important outcome of these projects. In this respect, Euro-BioImaging is at the forefront of these efforts by currently appointing a data steward to serve as a bridge between bioimaging researchers, communities, and repositories.

### Facet 2: Image data storage and management

As bioimaging technologies and associated datasets continue to evolve, the amount of data that is generated has grown exponentially. Today, individual experiments can easily produce terabytes of data. As a result, data management capabilities and strategies have typically failed to keep pace with the growth of image data size. It is therefore imperative for all imaging facilities and researchers to consider how data will be stored, documented, and moved during and after the project’s lifetime, before the data is even generated (Wallace et al. 2015; Schmidt et al. 2022).

As data volumes explode, conventional file-system-based storage solutions are quickly reaching their limits, partly because traditional microscopy data formats were not built for big data, let alone the cloud. As image data storage inevitably moves to cloud-based options, it is necessary to adapt image data formats to this mechanism to allow for open data access and sharing. The imaging community, organized by the Open Microscopy Environment (OME, (Swedlow et al. 2003)) is now addressing this need for a standardized file format by supporting and advancing Next Generation File Formats (NGFFs), specifically OME-Zarr, which is cloud-native and allows for easy streaming of chunks of very large datasets for interactive visualization and analysis (Moore et al. 2021, 2023).

Ensuring proper implementation of research data management (RDM) practices early in a project can significantly reduce the time for required data management and frustration that may arise later on. Having such a Data Management Plan (DMP) for research data is increasingly requested by funders, such as the European Commission,^10^ universities as well as research institutes. While the RDMkit^11^ compiles common guidelines to assist life scientists in their efforts to manage their research data, universities and facilities should also provide opportunities for training and consultation to scientists and personnel at various career stages. However, we must take care to ensure that the Data Management requirements do not become yet another administrative burden without value. To be of actual impact for the researcher, a DMP is thus properly tailored to the actual research project. Similar to the collection of metadata, the responsibility and effort of data management should not solely rest on the imaging scientists. Therefore, facilities and universities should provide and clearly communicate standardized data management procedures and recommendations. In addition, institutions could implement tools for data management plan creation such as the Data Stewardship Wizard (Pergl et al. 2019) or Argos^12^ that provide smart and tailored data management prompts for specific research projects. By currently offering data stewardship services, Euro-BioImaging assists its facilities by providing data management templates, and also individual researchers in adapting and customizing those templates to their particular project. Once vetted and further developed with the Euro-BioImaging community, a general version of these guidelines will be made openly available to the whole imaging community. In this way, life sciences Research Infrastructures are commonly shaping the current progress of data management recommendations. They achieve this through close contact with the needs of scientists as well as with funders and EU-funded consortia and cluster projects such as EOSC-Life^13^ as exemplified by the ongoing efforts of HE-funded projects such as ISIDORe^14^ to create umbrella DMPs (David et al. 2023). These extend into many research domains and encompasses research infrastructures beyond bioimaging which is particularly important to facilitate and manage cross-disciplinary projects.

Sensibly, data management strategies should not only consider the actual task of storing and moving the data, but also how the appropriate metadata is stored and maintained in conjunction with the actual data. By providing researchers with access to image data management platforms, such as OMERO (Allan et al. 2012), universities, institutions and facilities can increase the adoption and use of these tools. For example, the establishment of institutional OMERO servers is being catalyzed by the support and sharing of experiences through research infrastructures. This will result in FAIRer image data with minimal effort on the part of the researchers.

### Facet 3: Image analysis

Not only has the size of image data grown with the advances in resolution, speed, automation, and multimodality, but it has also become more complex and richer in information content. The analysis of such image data is hence more demanding, in terms of understanding the complex biological subject matter, the technological details of the microscopy method, as well as technical expertise and resources to efficiently deal with intricate image data in large volumes (Jamali et al. 2022). The visual nature of microscopy data has meant that in the past, analysis methods have been largely manually supported, making it hard to scale and report in a standardized manner. In fact, studies have shown that image analysis methods are vastly underreported in peer-reviewed studies (Marqués et al. 2020), thus hampering scientific rigor, reproducibility, and reusability. However, over time, there has been a growing emphasis on standardizing the reporting of image analysis methods (Schmied et al. 2023), even when the analysis is performed in a manual or semi-automated manner. In addition, we see a general shift towards more automated, modular, and thus reusable methodologies.

To take advantage of high performance and cloud computing it is necessary to support the current evolution of image analysis methods in making them more scalable, reproducible, and FAIR (Goble et al. 2020; Miura and Nørrelykke 2021). Following standardization, similar to other fields like genomics, image analysis methods are seeing increasing use of workflow management systems that allow users to chain different tools to create reproducible analysis workflows. They also promote shareability of analysis procedures by automatically storing the workflow in a machine-readable and executable format. At the same time, they enhance image data analysis in the big data sphere by facilitating parallelization and execution on high-performance computing resources and sometimes offer data storage and execution environments, hence enabling completely cloud-based analysis solutions. These platforms include generalized workflow management systems (Paul-Gilloteaux et al. 2021), such as Galaxy (Jalili et al. 2020), Nextflow (Di Tommaso et al. 2017), and Snakemake (Mölder et al. 2021) as well as platforms such as KNIME (Berthold et al. 2009) offering image data specific options for management, deployment and analysis that integrate different image-processing software programs. Moreover, generated workflows can be registered and thus disseminated in workflow registries such as the WorkflowHub (Goble et al. 2021). By adopting and extending common workflow management systems, Euro-BioImaging empowers life scientists to benefit from the European Open Science Cloud (EOSC^15^ ), an open multidisciplinary environment for hosting and processing research data to support EU science. In particular, we are building parallelized data conversion and analysis tools and workflows^16^ that assist researchers to remotely work with image data in the OME-Zarr format as part of the EOSC Future^17^ project, which aims to integrate existing data, resources, and services across scientific disciplines to provide a set of persistent and interoperable resources. Such efforts bridge the gap between image file formats and data repositories, thus further accelerating FAIR image data sharing.

The evolution in image analysis methodologies is largely driven by expert bioimage analysts (Miura 2016) that sit between the blurring boundaries of life and computer sciences domains. While larger research institutes and laboratories benefit from these dedicated personnel to meet their image analysis needs, small facilities and individual labs often lack the resources to host and fund such positions. To meet these growing analysis needs of researchers, Euro-BioImaging offers Image Data Analysis as a service, providing users with open access to expertise of bioimage analysts working at internationally recognized facilities and institutes at different Euro-BioImaging Nodes. By providing the possibility to decouple image data acquisition from analysis, researchers using this standalone service can leverage the expertise of specialized analysts who may be only available at distinct locations. On one hand, outsourcing the development of advanced and intricate analysis pipelines to experienced analysts allows researchers to make the most out of their image data thus increasing the overall output of a research project within its lifespan. On the other hand, it gives bioimage analysts the opportunity to work on external projects, increasing the impact radius of their expertise and their visibility beyond home institutions. This enhances the recognition of skilled personnel for both image analysis and image acquisition in facilities (Schlaeppi et al. 2022; Kivinen et al. 2022). Pioneering work in this direction has been done by the NEUBIAS community (Cimini et al. 2020) which brings together bioimage analysts from facilities distributed across Europe to define, streamline, and acknowledge the importance of image analysis in biological research. Through the NEUBIAS academy, they are actively working on providing training materials and courses, aimed at educating a new generation of bioimage analysts.

### Facet 4: Sharing of image data

Besides properly structured data and metadata, another hallmark of FAIR is sharing of research outputs beyond paper publication to the extent possible (Wilson et al. 2021). FAIR-advanced domains have established well-curated and deeply integrated repositories for certain data types (e.g., PDB,^18^ Genbank,^19^ UniProt^20^ ) and often the creation of these repositories leads to, or contributed at least to the success of, the development of new tools and even research areas (Feng et al. 2020; Swedlow et al. 2021). Over the last decade, the imaging community has witnessed the creation and more widespread adoption of bioimaging repositories and archives in an effort to establish a mature and functional bioimaging data ecosystem (Ellenberg et al. 2018). There are three key components to such an ecosystem: (1) primary archives, which allow for rapid and direct deposition of data associated with publications from a broad domain with a limited amount of metadata; (2) added-value databases that house curated data with rich metadata that are highly integrated with other resources, and (3) topic-specific resources or portals, bringing together data resources from different modalities and scientific domains, hosting, for example, organ-specific or biomedical data.

A central, primary archive for bioimaging data is currently provided by the BioImage Archive (BIA (Hartley et al. 2022)), which hosts data from all imaging modalities where a more specialized resource does not exist. To ensure minimal metadata quality, the BIA employs minimal metadata reporting according to REMBI guidelines. In contrast, the Image Data Resource (IDR (Williams et al. 2017)) is an added-value database for several light microscopic imaging modalities with highly curated metadata, including high-content screening data, aiming to link the imaging data with other databases, such as those for genetic and chemical information, as well as cell and tissue phenotypes. Public archiving of raw 2D electron microscopy data underlying 3D cryo-EM protein structures and 3D volume EM is performed by the Electron Microscopy Public Image ARchive (EMPIAR (Iudin et al. 2023)). The Systems Science of Biological Dynamics repository and database (SSBD (Tohsato et al. 2016)) are a pair of primary archive and added-value databases for quantitative data of spatiotemporal dynamics of biological objects mostly obtained from microscopy. According to the FAIR principles, data should be shared as openly as possible, but as closed as necessary. This principle is especially important for sensitive and biomedical data that cannot be fully openly shared and deposited. Thus, restricted-access and topic-specific repositories for bioimaging data include for example the Cancer Imaging Archive (Clark et al. 2013).

Given this variety of image repositories, it is important that the resources are well connected and coordinated, thus interoperable, to provide some level of consistency and thereby work towards creating a complete landscape of repositories to cover each type and modality of image data. Moreover, these data platforms will be most utilized when their deposition requirements and infrastructures are aligned with the current state of data production, not just data following tomorrow’s quality standards. Consequently, we believe it is crucial to initiate and maintain direct contact between depositors and archives, and are currently establishing this contact through the efforts of data stewards. This mutual exchange of real-world problems will meaningfully shape the deposition pipelines, thereby lowering barriers, time and energy required to easily share bioimaging data. In this way, data generation and deposition will evolve in parallel, leading to the production of increasingly FAIR data over time.

However, even the best and most thorough deposition ecosystem would be worthless if no data were deposited. Therefore, it is crucial to raise awareness of the FAIR image repositories, for example by engaging directly with researchers and institutions facilitated by data stewards and research infrastructures as a common platform. Yet, for widespread adoption of bioimaging data sharing, scientific journals have the leverage to shape standard practices by encouraging data sharing and providing targeted suggestions for repositories. As a step in the direction of FAIR science, they are increasingly requiring data availability statements to be included in the publication as well as directing authors to resources like FAIRsharing (Sansone et al. 2019), a catalog of databases, standards, and policies, that can help to discover suitable repositories. Infrastructures and communities are in active communication with journal representatives to define clearer incentives and recommendations for image-sharing platforms and procedures. In addition, permissive data availability should be more rewarded thereby highlighting and promoting well-documented and reproducible studies. Many ecosystem partners invest considerable effort to raise awareness of the additional benefits of data sharing such as increased recognition of scientific effort beyond papers (Wilson et al. 2021) including increased citations (Colavizza et al. 2020) and reduced burden of long-term data storage (Ellenberg et al. 2018). The popularity and usage of image repositories will increase as a result of these efforts. Our vision is to move to a place where data sharing alongside publication is as second nature for bioimaging data as it is in other fields.

### Facet 5: Establishing trust in data to foster reuse

The ultimate goal of the FAIR principles is to increase the value of scientific data by promoting its reuse in subsequent studies, thus getting more out of the effort expended to generate the data in the first place. In theory, if data is available in archives, data reuse should be possible. However, we currently see very little reuse of bioimaging data; in fact, there is a large discrepancy between bioimaging researchers who believe they could benefit from data reuse and those who actually incorporate image reuse into their research (Schmidt et al. 2022). Reasons for this discrepancy may be a lack of awareness of other datasets, so there is a need to spotlight well-documented datasets with rich information content. But even if the data can be located and retrieved, reuse requires that the data is of adequate quality, because data of questionable quality can be challenging to interpret and should not be reused (Miura and Nørrelykke 2021). Indeed, a recent study in the fields of ecology/evolution found that nearly two-thirds of the data in public archives in these domains were "unusable" (Roche et al. 2015). Given this, it is not surprising that scientists are generally skeptical towards other researchers' data (Chan et al. 2021), which manifests itself as a reluctance to reuse deposited data. Thus, as it did in other disciplines like structural biology, the establishment of common data and metadata standards will also increase confidence in the deposited data and foster reuse. Community initiatives dedicated to data quality such as QUAREP-LiMi for Light Microscopy and the volumeEM community,^21^ play a crucial role in the development and adoption of such standards while also underpinning them with the required broad support and credibility. Bioimaging data archives and databases are currently engaging with the community to work towards implementing metadata reporting standards as described above while also exploring mechanisms to evaluate and declare the quality of data reporting, thus highlighting well-documented datasets. In the future, it will also be valuable to implement and display internal quality controls of the image data itself and the associated imaging experiment, as is the case with structural biology data.

Additionally, reuse of image data will be enhanced by promoting the findability of image data from other life science domains by providing concrete and relevant cross-links between publicly available image data and other data types through curation. Several of these interdisciplinary communication approaches, such as 3DBionotes-WS,^22^ a web service focusing on annotating structural models with relevant biochemical and biomedical information, and the COVID-19 disease map (Ostaszewski et al. 2021), are now working towards the integration of bioimaging data.

Another important factor in facilitating reuse and increasing the credibility of data is that the license for reuse must be properly defined and the provenance of the data must be clear (Huisman et al. 2019; Carbon et al. 2019). Only when people are aware that they can use the data safely, and know exactly where it came from and how it was created, can they use it properly. Especially for sensitive data, it is important to clarify how, to what extent, for what purpose, and in what context others are allowed to reuse the data (Navale and McAuliffe 2018). Euro-BioImaging, also in the context of European projects like BY-COVID and EUCAIM,^23^ is involved in defining guidelines for both best practices and the ethics of sharing and reuse of these types of data. Furthermore, to encourage and normalize the increasingly widespread reuse of bioimaging data, it is imperative to credit data owners when their datasets are reused, so that they get attributed for their scientific work beyond traditional publications (Pierce et al. 2019). This approach directs towards a future where the most important scientific output of researchers is not just the individual contributions to papers, but also the totality of datasets, pipelines, software, and protocols of value to the scientific community that they have produced.

In summary, progress is needed on all of the above-described facets of FAIR data to collectively increase the volume and quality of openly available bioimages, in order to build trust and thereby encourage reuse. Currently, however, the perceived value of archived datasets is still low, so we still lack a community that pioneers data reuse and favors reuse over new data generation. Recently, the artificial intelligence (AI) community has begun to emphasize the benefits of sharing bioimaging data since deep learning algorithms need to be trained on large, diverse, and well-annotated datasets (Ouyang et al. 2022). This reinforces the need to prioritize data sharing and standardized metadata for the development of advanced AI algorithms for bioimage analysis. In this regard, Euro-BioImaging is coordinating the European project AI4Life,^24^ which aims to bridge the gap between computer and life sciences communities by offering infrastructure and services to support life scientists in adopting machine learning solutions for bioimage analysis. Ongoing discussions within AI4Life focus on developing annotation standards and improving the submission, presentation, and retrieval of AI-ready images and annotations. To fully leverage the potential of AI, it is crucial to ensure that the metadata associated with both the AI models and the data used to train them are of high quality. Standardized metadata is therefore essential for effective data exploitation and reuse, and a priority for the scientific community.

### Facet 6: Dissemination of FAIR practices

The mostly technical facets of FAIR data described above cannot be considered as separate entities, but must be treated as interdependent and interrelated. Progress in one area advances the others, and conversely, lack of progress in one area hinders advances in others. Therefore, careful consideration of their intersections is necessary to promote progress in all facets simultaneously. Additionally, the imaging community is very diverse and highly engaged in this topic, leading to the formation of a number of different community initiatives dedicated to various aspects of the image data challenge. These initiatives are all individually highly valuable, but to ensure efficiency of the mostly volunteer work involved in these, facilitating their alignment with each other and with science policy is critical. Thus, coordination, integration, and communication at the regional, national, and global levels are equally necessary to successfully integrate FAIR image data initiatives into a landscape of independent community initiatives and science policy interests.

This is a key priority and responsibility of large-scale research infrastructures like Euro-BioImaging which have the necessary connections within the landscape and have acquired an in-depth understanding of science policy developments. With the creation of its Image Data Expert Group,^25^ Euro-BioImaging has become a common discussion platform for image data service provision, and consolidation and discussion on current developments by community initiatives. Owing to its vast network of national and institutional facilities that participate in National data and RDM initiatives, Euro-BioImaging is uniquely positioned to facilitate interactions between them, and promote coordinated development instead of duplication of efforts. Additionally, great emphasis is placed on disseminating the progress of the Horizon Europe funded projects that we participate in and their practical results relevant to the European image data communities. These projects are currently providing critical resources for the technical facets of the FAIR bioimaging data as well as community support. In the future, further relevant EU projects will increase the presence of bioimaging data in the European Health Data Space of digital infrastructures dealing with the management and re-use of clinical data.

The overall expected outcome of these European-level projects is to facilitate the participation and contribution of the European image data community to the EOSC, a key strategic priority of the European Commission contributing to the European Digital Decade. European researchers, but also innovators, businesses and citizens, can publish, discover, and reuse data, tools and services for research, innovation, and education within the federated and open multidisciplinary environment of EOSC. By proactively engaging with the EOSC, Euro-BioImaging's image data strategy and services contribute to and align with EOSC developments thus allowing researchers to take full advantage of cloud services. This creates and fosters the impact of bioimaging in FAIR big data research on the European landscape and beyond.

However, successful plans for image data sharing cannot be restricted to one continent. To this end, the Global BioImaging^26^ network was established as an exchange platform for staff and managers working in national and regional imaging infrastructures and communities representing currently 27 countries around the world.^27^ Here, international working groups such as the one for Image Data Management convene to ensure that open discussion on data formats and standardization takes place internationally, and communities can align their efforts globally (Swedlow et al. 2021). Euro-BioImaging is the launching partner and a key stakeholder in Global BioImaging, working closely together on topics like biological and preclinical image data, staff training, career development, funding, and policy. These interactions and exchanges on the global level bring the international imaging community closer together to make FAIR image data a truly global resource.

## Conclusions

As the volume and potential of biological images grows, everyone from individual researchers and institutions to funding agencies and the general public has expectations regarding image and metadata quality, accessibility, and potential for analyzing the resource. Following the growing adoption of Open Science policy and the FAIR principles for scientific data, the need for image data services is growing rapidly. Together with other national and international imaging initiatives, Euro-BioImaging has set out to tackle these challenges and is helping the research community to collectively develop much-needed solutions for managing, sharing, and analyzing image data. Each member of the FAIR imaging ecosystem influences the area in which they are most engaged and involved, but the entire ecosystem has to work together to impact the advancement of the field as a whole. Once the combined efforts described by community members in this Special Edition are successfully and widely adopted, we are anticipating a drastic shift in the way images are used, shared, and reused in biology. In the future, new research studies will build on re-analysis of existing image data and cross-references between already-existing datasets rather than generating new data. In this way, image data will become a global resource that everybody can use beyond national boundaries, creating research opportunities in many places which are still lacking imaging technologies for data acquisition. As a whole, more readily available image data will also be a catalyst for greater public interest and trust in science. These efforts echo the unprecedented opportunities of today’s imaging technologies in the life sciences, and the need for a predictive understanding of biology, with pandemics and the effects of climate change on our ecosystems increasingly affecting our everyday life.

## Data Availability

Data sharing is not applicable to this article as no datasets were generated or analyzed.

## Footnotes

1 https://research-and-innovation.ec.europa.eu/strategy/strategy-2020-2024/our-digital-future/european-research-infrastructures_en.

2 https://www.eurobioimaging.eu/.

3 https://www.springer.com/journal/418/submission-guidelines.

4 https://www.embopress.org/page/journal/14602075/authorguide.

5 https://www.eurobioimaging-industryboard.com/.

6 https://by-covid.org/.

7 https://eosc4cancer.eu/.

8 https://www.w3.org/TR/vocab-dcat-3/.

9 https://bioschemas.org.

10 https://ec.europa.eu/research/participants/docs/h2020-funding-guide/cross-cutting-issues/open-access-data-management/data-management_en.htm.

11 https://rdmkit.elixir-europe.org/.

12 https://argos.openaire.eu/home.

13 https://www.eosc-life.eu/.

14 https://isidore-project.eu/.

15 https://eosc-portal.eu/.

16 https://github.com/Euro-BioImaging.

17 https://eoscfuture.eu/.

18 https://www.rcsb.org/.

19 https://www.ncbi.nlm.nih.gov/genbank/.

20 https://www.uniprot.org/.

21 https://www.volumeem.org.

22 https://3dbionotes.cnb.csic.es/ws.

23 https://digital-strategy.ec.europa.eu/en/policies/cancer-imaging.

24 https://ai4life.eurobioimaging.eu/.

25 https://www.eurobioimaging.eu/about-us/expert-groups#third.

26 https://globalbioimaging.org/.

27 Advanced Bioimaging Support (Japan), BioImaging North America, Canada BioImaging, Euro-BioImaging, India BioImaging, Latin America BioImaging, Laboratorio Nacional de Microscopía Avanzada (Mexico), Microscopy Australia, National Imaging Facility (Australia), SingaScope (Singapore) and South Africa BioImaging.
